# Turmeric powder and its derivatives from *Curcuma longa* rhizomes: Insecticidal effects on cabbage looper and the role of synergists

**DOI:** 10.1038/srep34093

**Published:** 2016-11-02

**Authors:** Wagner de Souza Tavares, Yasmin Akhtar, Gabriel Luiz Padoan Gonçalves, José Cola Zanuncio, Murray B. Isman

**Affiliations:** 1 Departamento de Fitotecnia, Universidade Federal de Viçosa, 36570-900, Viçosa, Minas Gerais, Brazil; 2 Faculty of Land and Food Systems, University of British Columbia, Vancouver, BC, V6T 1Z4 Canada; 3 Departamento de Entomologia e Acarologia, Universidade de São Paulo, 13418-900, Piracicaba, São Paulo, Brazil; 4 Departamento de Entomologia/BIOAGRO, Universidade Federal de Viçosa, 36570-900, Viçosa, Minas Gerais, Brazil

## Abstract

*Curcuma longa* has well-known insecticidal and repellent effects on insect pests, but its impact on *Trichoplusia ni* is unknown. In this study, the compound *ar*-turmerone, extracted and purified from *C. longa* rhizomes, was identified, and its insecticidal effects, along with turmeric powder, curcuminoid pigments and crude essential oil were evaluated against this important agricultural pest. The role of natural (sesamol and piperonal) and synthetic [piperonyl butoxide (PBO)] synergists under laboratory and greenhouse conditions were also evaluated. The concentration of *ar*-turmerone in *C. longa* rhizomes harvested was 0.32% (dwt). Turmeric powder and its derivatives caused 10–20% mortality in third instar *T. ni* at a very low dose (10 μg/larva). Addition of PBO increased toxicity of turmeric powder and its derivatives (90–97% mortality) in most binary combinations (5 μg of turmeric powder or its derivatives +5 μg of PBO), but neither piperonal nor sesamol were active as synergists. The compound *ar*-turmerone alone and the combination with PBO reduced larval weight on treated *Brassica oleracea* in the laboratory and in greenhouse experiments, compared with the negative control. The compound *ar*-turmerone could be used as a low cost botanical insecticide for integrated management of cabbage looper in vegetable production.

Plants produce toxic substances as natural defenses against pests, such as insects and pathogens^1^,^2^. These substances can be extracted from plants and used in the production of commercial insecticides^3^,^4^. The use of plants, plant material (bark, leaves, roots, seeds, and stem) or crude plant extracts as sources of insecticidal substances for crop protection began at least two millennia ago and are being used today in organic farming^5^. Traditionally used botanical insecticide products include nicotine, rotenone, ryania, sabadilla, and pyrethrum^5^. Rotenone, derived from tropical legumes in the genera *Derris* Lour. and *Lonchocarpus* Kunth (Fabales: Fabaceae), and ryania, derived from the South American shrub *Ryania speciosa* Vahl. (Malpighiales: Salicaceae), are used against horticultural and ornamental crop pests. Pyrethrum, extracted from flowers of Dalmatian chrysanthemum, *Tanacetum cinerariifolium* (Trevir.) Sch. Bip. (Asterales: Asteraceae), is the most important botanical insecticide on the market. Pyrethroids (deltamethrin, permethrin, tetramethrin, etc.) are synthetic derivatives of the natural pyrethrins and have enjoyed enormous commercial success as crop protectants and in other areas of pest management. Both natural pyrethrin and synthetic pyrethroid-based products usually contain a synergist such as piperonyl butoxide (PBO) or piperonyl cyclonene that substantially increases the efficacy of the active ingredient and reduces its cost^6^. PBO enhances pyrethrin activity about 4-fold through the inhibition of detoxicative cytochrome P450 enzymes^7^.

The toxicity of natural substances to insect pests can sometimes be comparable to that of synthetic insecticides, but often with less environmental effects^4^,^8^. Evaluation of toxicity in the laboratory against the target pest is the first step toward developing a commercially available insecticide^9^,^10^. The next step is to determine efficacy under more realistic conditions, such as in a greenhouse or a small field plot^11^,^12^. Natural products recommended for organic agriculture tend to be safer alternatives to conventional insecticides^13^,^14^ owing to their rapid degradation, lack of persistence, and minimal adverse effects on humans, beneficial insects and environment^5^.

Turmeric, *Curcuma longa* L. (Zingiberales: Zingiberaceae) (synonym: *Curcuma domestica* Valeton), an herbaceous perennial plant with long lateral ramifications, originated from Southeast Asia, probably in the Indian subcontinent^15^,^16^. Turmeric powder is extracted from the dried ground rhizomes of this plant for culinary uses^17^,^18^. Natural products from this plant also have analgesic, antibacterial, antifungal, anti-inflammatory, antioxidant, and digestive properties^19^,^20^ and are under investigation as possible treatments for Alzheimer’s disease, arthritis, diabetes, cardiovascular, liver and kidney problems, several types of cancer, and other clinical disorders^21^,^22^. The fresh juice, alcoholic and aqueous extracts, and essential oils of *C. longa* have demonstrated insecticidal effects against a number of insect pests, and also repelled mosquitoes^13^,^23^,^24^,^25^. *Curcuma longa* is harvested when the aerial part of this plant senesces after flowering and its rhizomes develop an intense yellow color indicating the presence of concentrated pigments^26^,^27^.

Insecticidal substances can be obtained from *C. longa* and other plant species, in low concentrations. For example, 0.32% and 0.50% (dwt) *ar*-turmerone was extracted from *C. longa* rhizomes in Brazil^13^ and aerial parts of black mint, *Tagetes minuta* L. (Asterales: Asteraceae) in Kenya^28^, respectively. Essential oil of the rhizomes of *C. longa* produced 44.4% and 38.6% (dwt) *ar*-turmerone in Nigeria^29^ and Pakistan^30^ respectively, whereas 63.4% (dwt) *ar*-turmerone was extracted from the essential oil of leaves in Nigeria^29^.

Climatic and genetic factors, harvesting time, soil type, fertilization, drying process, and period of storage can all affect the chemical composition of essential oils from *C. longa*^30^,^31^,^32^. The composition and volatility of *C. longa* essential oils determine the characteristic smell of turmeric, whereas fixed phenolic compounds, such as the pigment curcumin (a diarylheptanoid), its derivatives and other substances, are responsible for the intense yellow color of the rhizomes^33^. Volatile essential oils of *C. longa* contain a mixture of ketones and sesquiterpene alcohols, the latter mainly based on germacrene and bisabolane skeletons^34^,^35^.

The cabbage looper, *Trichoplusia ni* (Hübner, 1800–1803) (Lepidoptera: Noctuidae) (synonyms: *Phytometra brassicae* and *Plusia innata* Herrich-Schaffer, 1868), a serious pest of cruciferous crops, is found throughout the southern Palaearctic ecozone, including all of North America, parts of Africa, much of eastern Europe, and the Indo-Australian region^36^. This species is very destructive to plants due to its voracious consumption of foliage^37^,^38^. *Trichoplusia ni* is not restricted to cabbage, *Brassica oleracea* L. (Brassicales: Brassicaceae), but also damages cucumber (*Cucumis sativus* L., Cucurbitales: Cucurbitaceae), potato (*Solanum tuberosum* L.) and tomato (*Solanum lycopersicum* L.) (Solanales: Solanaceae)^8^. Because *T. ni* has evolved resistance against many synthetic insecticides and the microbial insecticide *Bacillus thuringiensis* Berliner (Bacillales: Bacillaceae)^6^, it is important to develop new tools or materials that could be used to protect crops compatible with integrated pest management (IPM) schemes^7^.

The objective of the current study was to identify insect bioactive compounds, extracted and purified from *C. longa* rhizomes, and to determine their insecticidal effects, comparing bioactivity among *ar*-turmerone, turmeric powder, curcuminoid pigments, and a crude essential oil using *T. ni* as the test organism. We also assessed the role of natural (sesamol, piperonal) and synthetic (PBO) synergists under laboratory and greenhouse conditions. Synergists were added to enhance the efficacy of turmeric powder and its derivatives against the cabagge looper. Natural and synthetic synergists such as sesamol, piperonal and PBO inhibit detoxifying enzymes in insects and other organisms^39^,^40^ thus enhancing insecticide efficacy^41^,^42^. Synergists can also delay resistance development in insects^43^. For example, the speed of selection for deltamethrin (a synthetic pyrethroid insecticide) resistance was reduced by 60% in susceptible yellow fever mosquito, *Aedes aegypti* (Linnaeus *in* Hasselquist, 1762) (Diptera: Culicidae) larvae subjected to a mixture of deltamethrin and PBO in the ratio of 1:5 for 20 generations^44^. A pyrethrin-based insecticide was selected as a positive control because we wanted to compare the efficacy of our treatments with a commercial botanical insecticide. Schulz Insect Spray^®^ contains 0.02% pyrethrins as the active ingredient and 0.20% PBO as a synergist. Schultz Insect Spray^®^ is recommended for use on indoor and outdoor plants, including edible vegetables, and is a broad-spectrum insecticide.

## Results and Discussion

### Yield of *ar*-turmerone

The yield of *C. longa* essential oil with hexane was 0.39% (dwt) (1.93 g) and that of *ar*-turmerone from this essential oil was 82% (dwt) after the chromatographic separations from the initial material (1.58 g). A total of 3.2 g of *ar*-turmerone was present per kg of rhizomes of this plant grown in Catalão, Goiás, Brazil. Consistent with our results, high concentrations of *ar*-turmerone in non-polar extracts and essential oils of *C. longa* have been reported from China (Asia), India (Asia), Nigeria (Africa), Pakistan (Asia), and the islands of Sao Tome and Principe (Africa)^29^,^45^,^46^,^47^. The quantitative and qualitative compositions of plant extracts and essential oils depend on genetic factors and on the environmental conditions of the area where the plant is grown, with variations in the essential oils of *C. longa* occurring at different localities^30^,^31^,^32^. *Curcuma longa* can be cultivated at low cost and sustainably in Brazil using minimal labour, a range of growing seasons and spacing, and organic fertilization with 50 tons of cattle manure per ha^48^.

### Contact toxicity (topical application) of turmeric powder and derivatives on cabbage loopers in the laboratory

The initial screening dose (10 μg/larva) demonstrated low toxicity through topical application to third instars, ranging from 10–20% at 24 h (Table 1). Binary mixtures with piperonal as a synergist slightly improved toxicity of treatments in some cases (turmeric powder + piperonal and *ar*-turmerone + piperonal). Sesamol did not enhance activity in any treatment. However, addition of PBO as a synergist increased toxicity of turmeric powder and all of its derivatives.

Mortality caused by exposure of cabbage looper larvae to binary mixtures of turmeric crude essential oil + PBO, turmeric powder + PBO and *ar*-turmerone + PBO were 90%, 94% and 96%, respectively, even though the dose of the active ingredient was reduced by one-half. Mortality caused by binary mixtures of curcuminoid pigments and PBO was almost twofold that of the individual compounds. The positive control (Schulz Insect Spray^®^), tested at 1%, produced 71.4% mortality (Table 1). Similar to our study, a 1% (m · v^−1^) acetonic solution of *ar*-turmerone mixed in an artificial diet produced 58.3% mortality of one-day-old larvae of the fall armyworm, *Spodoptera frugiperda* (Smith, 1797) (Lepidoptera: Noctuidae) after 10 days of feeding^13^. The compound *ar*-turmerone caused 100 and 64% mortality of adult brown planthopper, *Nilaparvata lugens* (Stål, 1854) (Hemiptera: Delphacidae) females at 1,000 and 500 ppm, respectively. Against larvae of the diamondback moth, *Plutella xylostella* (Linnaeus, 1758) (Lepidoptera: Plutellidae), the compound produced 100 and 82% mortality at 1,000 and 500 ppm, respectively. Against green peach aphid, *Myzus persicae* (Sulzer, 1776) (Hemiptera: Aphididae) adult females and tropical armyworm, *Spodoptera litura* (Fabricius, 1775) (Lepidoptera: Noctuidae) larvae, *ar*-turmerone at 2,000 ppm was effective but demonstrated weak insecticidal activity at 1,000 ppm. At a dose of 2.1 mg · cm^−2^, *ar*-turmerone was almost ineffective (<10% mortality) against rice weevil adults, *Sitophilus oryzae* (Linnaeus, 1763) (Coleoptera: Curculionidae), cowpea bruchid, *Callosobruchus chinensis* (Linnaeus, 1758) (Coleoptera: Chrysomelidae) and tobacco beetle, *Lasioderma serricorne* (Fabricius, 1792) (Coleoptera: Anobiidae) as well as larvae of the Indian mealmoth, *Plodia interpunctella* (Hübner, 1813) (Lepidoptera: Pyralidae)^49^. An artificial diet treated with acetonic solutions of extracts of *C. longa* rhizomes fed to the freshly emerged peach fruit flies, *Bactrocera zonata* (Saunders, 1841) (Diptera: Tephritidae) for 16 days at 1,000, 500 and 250 ppm produced 84.7, 79.0 and 67.7% mortality, respectively. Eggs deposited by the surviving females on clean guava fruits (*Psidium guajava* L., Myrtales: Myrtaceae) in separate cages demonstrated 67.9, 60.7 and 51.9% pupal inhibition for the flies fed on 1,000, 500 and 250 ppm of *C. longa* extracts respectively^50^.

### Dose response effects of turmeric powder and derivatives on cabbage loopers in the laboratory through contact toxicity (topical application)

Based on the LD_50_ values (Table 2), binary mixtures of turmeric powder + PBO (LD_50_ = 0.03 μg) and turmeric crude essential oil + PBO (LD_50_ = 0.05 μg) were both significantly more toxic than *ar*-turmerone + PBO (LD_50_ = 0.26 μg) which itself was more toxic than curcuminoid pigments + PBO (LD_50_ = 0.61 μg) against third instar cabbage loopers (based on non-overlapping 95% confidence intervals) (Table 2). PBO synergized toxicity of turmeric compounds to the cabbage looper, as previously reported for alpha-cypermethrin (a synthetic pyrethroid insecticide) and xanthotoxin (a furanocoumarin-type plant natural product) 72 h after exposure to the navel orangeworm, *Amyelois transitella* (Walker, 1863) (Lepidoptera: Pyralidae)^39^. PBO is well recognized to enhance the action of pyrethroids and organophosphates in the malaria mosquito, *Anopheles gambiae* Giles, 1902 (Diptera: Culicidae)^40^, and deltamethrin in a pyrethroid-resistant strain of *A. aegypti*^41^.

### Growth inhibitory effects of turmeric powder and its derivatives on cabbage loopers in the laboratory through feeding

Weight of cabbage loopers reared on artificial diets incorporating turmeric powder or its derivatives and their binary mixtures with PBO after seven days in the laboratory showed a significant effect (one-way ANOVA; F_13,105_ = 5.5; p < 0.05). Weights of larvae were significantly lower than negative controls for all treatments (Tukeys’ test; p < 0.05). Larval weight was significantly lower on *ar-*turmerone (119.1 mg), *ar-*turmerone + PBO (81.8 mg) and *ar-*turmerone + sesamol (116.7 mg) treatments, compared to all other treatments including the negative control (297.8 mg) and the positive control (200.0 mg). Weight reduction for these treatments varied from 60 to 72% compared with the negative control (Table 3). The positive control reduced growth by 35.2% at 1,000 ppm (Table 3). Red flour beetle, *Tribolium castaneum* (Herbst, 1797) (Coleoptera: Tenebrionidae) adults, fed on wheat flour (*Triticum aestivum* L., Poales: Poaceae), which had been treated with turmeric oil at 200 ppm produced fewer and underweight larvae, pupae, and adults compared with those fed on untreated flour^51^. Curcuminoids, comprising three closely related curcumins (I, II, and III) of turmeric rhizome powder, were screened for their growth inhibitory activity against the desert locust, *Schistocerca gregaria* (Forsskål, 1775) (Orthoptera: Acrididae) and the red cotton bug, *Dysdercus koenigii* (Fabricius, 1775) (Hemiptera: Pyrrhocoridae) nymphs. At a dosage of 20 μg per *S. gregaria* fifth instar nymph, curcumins injected into the hemolymph, produced 40–50% growth inhibition and 10–15% mortality. Turmeric oil produced 10% growth inhibition and 60% nymphal mortality at the same dosage. Topical application of a dosage of 50 μg of curcuminoids (I, II, and III) produced 45% growth inhibition of *D. koenigii* nymphs^52^.

Our study demonstrated that dietary EC_50_ values were lowest for *ar*-turmerone (608.7 ppm) followed by turmeric powder (765.3 ppm) and turmeric crude essential oil (844.4 ppm). The EC_50_ values for the binary mixtures of *ar*-turmerone + PBO were also the lowest (304.7 ppm), followed by turmeric powder + PBO (471.2 ppm) and the binary mixture of crude essential oil and PBO (598.9 ppm) (Table 4). Similar level of synergy has been reported in mixtures of turmeric oil and azadirachtin, a triterpenoid from *Azadirachta indica* A. Juss. (Meliales: Meliaceae) against third/fifth instar of the Bihar hairy caterpillar, *Spilosoma obliqua* (Walker, 1855) (Lepidoptera: Arctiidae). Among the three different ratios (1:1, 2:1, and 3:1) of azadirachtin-turmeric oil mixture, 1:1 mixture exhibited pronounced insect growth regulator (IGR) activity (EC_50_ = 1.26 × 10^−2^/2.16 × 10^−2^%) and considerable antifeedant activity (EC_50_ = 2.90 × 10^−2^/1.0 × 10^−2^%) against *S. obliqua* larvae^53^.

### Protection of cabbage leaves treated with turmeric powder and derivatives with or without synergists in the laboratory

Weight of cabbage loopers reared on cabbage leaves treated with turmeric powder or its derivatives and their binary mixtures with PBO for four days in the laboratory showed a significant effect (one-way ANOVA; F_7,122_ = 4.9; p < 0.05). Larval weight was significantly lower on *ar*-turmerone (24.3 mg) and *ar*-turmerone + PBO (14.5 mg) treatments compared with the negative control (46.1 mg) (Tukeys’ test; p < 0.05). Larval weights were reduced by 47 and 69% respectively in the *ar*-turmerone and *ar*-turmerone + PBO treatments, compared with the control. Similarly, the number of larvae recovered from the leaves was also the lowest on these two treatments (Table 5). Larval recovery from the leaves treated with *ar*-turmerone + PBO was only 36% relative to the control. Reductions in weight and number of cabbage loopers recovered from cabbage leaves treated with *ar*-turmerone or *ar*-turmerone + PBO are similar to those seen with the cotton bollworm, *Helicoverpa armigera* (Hübner, 1805) (Lepidoptera: Noctuidae) larvae. First instar *H. armigera* were reared on a semi-synthetic diet treated with 5% of *C. longa* rhizome powder for 7–10 days. Larval and pupal weights, survival, development time, and adult emergence rate were adversely affected by *C. longa* treatments. There was 69% growth inhibition in larvae and the adult emergence period was prolonged by 8 days compared with the control^54^. Turmeric extracts have been shown to protect stored wheat, and increase egg mortality in the Angoumois grain moth, *Sitotroga cerealella* (Olivier, 1789) (Lepidoptera: Gelechiidae) when treated with 1,000 ppm of turmeric extracts prepared in acetone, ethanol or petroleum ether^23^.

### Protection of intact cabbage plants treated with turmeric powder and derivatives in the greenhouse

**Figure 1:**
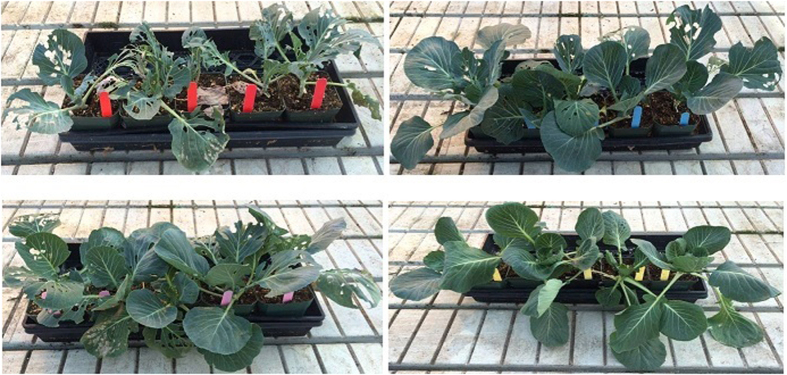
Cabbage, *Brassica oleracea* var. Stonehead (Brassicaceae) plants infested with third instar cabbage looper, *Trichoplusia ni* (Lepidoptera: Noctuidae) three days after spraying with *ar*-turmerone from *Curcuma longa* (Zingiberaceae) +/− piperonyl butoxide (PBO). Acetone alone was used as a negative control and Schultz Insect Spray^®^ (0.02% pyrethrins + 0.20% PBO) as a positive control. One plant was cultivated per plastic pot. Five plastic pots were used per plastic tray (15 plants per treatment). Top, left–negative control; top, right–*ar*-turmerone; bottom, left–positive control; bottom, right–*ar*-turmerone + PBO.

Greenhouse results (Table 6, Fig. 1) were consistent with those observed in the laboratory. Cabbage looper larvae weighed significantly less on *ar*-turmerone (15.1 mg) and *ar*-turmerone + PBO (14.6 mg) treatments compared with the negative control (49.5 mg). Weight of cabbage loopers reared on cabbage leaves treated with turmeric powder, its derivatives and their binary mixtures with PBO for four days showed a significant effect (one-way ANOVA; F_7,180_ = 11.8; p < 0.05). There was a 69.5 and 70.0% reduction in weight of the larvae on *ar*-turmerone and *ar*-turmerone + PBO treated plants, respectively compared with the negative control (Tukeys’ test; p < 0.05). Although larval weights were lowest on *ar-*turmerone and *ar*-turmerone + PBO, they did not differ significantly from turmeric crude essential oil + PBO (22.6 mg). Larval weight was significantly lower on turmeric crude essential oil + PBO (22.6 mg) compared with the crude essential oil (36.9 mg) alone. Numbers of larvae recovered from these treatments were also the lowest (Table 6). Larval recovery from cabbage plants treated with *ar*-turmerone + PBO was only 38% relative to the control. Results in the greenhouse confirm the toxicity of *ar*-turmerone (+/−PBO) and although not insecticidal in some cases, it can suppress larval growth and reduce feeding damage caused by this pest. Larvae from the *ar*-turmerone treatment (+/−PBO) were significantly lighter (~70% reduction in weight) and weaker than the control, increasing their probability of being preyed upon by natural enemies, as suggested for *S. frugiperda* larvae fed on an artificial diet treated with an acetonic solution of *ar*-turmerone^13^.

## Conclusion

An insecticide based on turmeric powder or some of its derivatives, especially the sesquiterpene *ar*-turmerone, could potentially control the cabbage looper larvae. In contrast, curcuminoid pigments were not active. Addition of PBO increased efficacy of turmeric solutions in most combinations, whereas the natural products, piperonal and sesamol, were not synergistic. The *ar*-turmerone could be a low-cost and sustainable alternative for IPM of cabbage looper larvae and the addition of PBO can improve its efficacy. Since the treatments exhibit more than one mode of action, it is believed that this will delay resistance development in cabbage looper and other insects. Plant defense chemicals that attack pests at multiple levels are especially suitable for crop protection.

## Methods

### Plant material

Rhizomes of *C. longa* were collected from a commercial crop grown on the Macaúba farm in Catalão, Goiás, Brazil (18° 08′S, 47° 57′W, 515 m above sea level). Five large plants, free of pests or diseases, were chosen within the crop. The soil was dug with a hoe until rhizomes became visible. Entire plants were harvested and the rhizomes were cut from the plants with a knife. The rhizomes were placed in a polystyrene box lined with ice and brought to the laboratory, where they were washed in running water, dried and stored at 2 °C. This farm uses no synthetic agrochemicals.

### Chemicals

**Figure 2:**
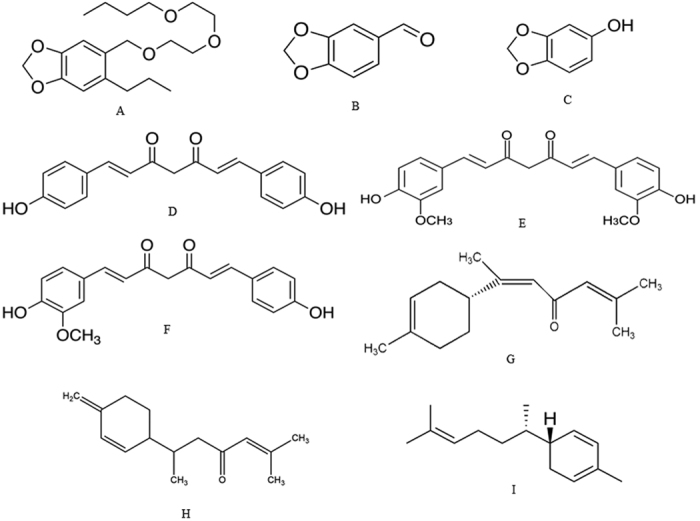
Chemical structures of the synergists piperonyl butoxide (PBO) (synthetic) (**A**), piperonal (**B**) and sesamol (naturals) (**C**); major components of turmeric powder and curcuminoid pigments: bisdemethoxycurcumin (**D**), curcumin (**E**) and demethoxycurcumin (**F**) and of volatiles of crude essential oil from *Curcuma longa* (Zingiberaceae) rhizomes: α-atlantone (**G**), β-turmerone (**H**) and zingiberene (**I**).

Schultz Insecticide, Houseplant & Indoor Garden Insect Spray^®^ (Premier Tech Home & Garden Inc., Brantford, Canada) was used as the positive control. It contains 0.02% pyrethrins as the active ingredient and 0.20% PBO as a synergist (Fig. 2A)^55^. Piperonal (C_8_H_6_O_3_) (aka heliotropin) and sesamol (C_7_H_6_O_3_) (aka 3,4methylenedioxyphenol or 1,3-benzodioxol-5-ol) were used as natural synergists. Piperonal is a compound commonly found in fragrances and flavors. It is structurally related to other aromatic aldehydes such as benzaldehyde and vanillin. Piperonal naturally occurs in various plants, including dill (*Anethum graveolens* L., Apiales: Apiaceae), violet flowers (*Viola odorata* L., Malpighiales: Violaceae) and black pepper (*Piper nigrum* L., Piperales: Piperaceae) (Fig. 2B). Sesamol is a natural component of sesame oil, which is an edible oil derived from sesame seeds (*Sesamum* spp., Lamiales: Pedaliaceae). It can also be produced via synthesis from heliotropine (Fig. 2C)^56^,^57^. PBO was used as a synthetic synergist. All synergists were purchased from Sigma-Aldrich (Canada) and their purity varied from 98 to 100%.

### Experimental procedures

^1^H, ^13^C, Heteronuclear Single Quantum Coherence (HSQC), and Heteronuclear Multiple Bond Correlation (HMBC) Nuclear Magnetic Resonance (NMR) measurements were carried out on a Bruker Avance III 500 instrument (operating at 500.13 MHz for ^1^H) equipped with a 5 mm triple Resonance broadband inverse probehead (TBI) with Z-gradient. Deuterated chloroform was used as solvent and tetramethylsilane as the internal standard. Mass spectra were obtained by gas chromatography coupled to a mass spectrometry (GC-MS). The GC-MS analyses were performed using a gas chromatograph [GC-17A Shimadzu, GC-MS/QP5,000 Shimadzu, DB-5 column (30 mm × 0.32 mm)], with ionization by electronic impact, under the following conditions: 60 °C for 3 min; 5 °C · min^−1^ to 240 °C, for 8 min; with an injector temperature of 180 °C, a detector temperature of 260 °C and an injection volume of 1-L. Mass spectra were compared with the National Institute of Standards and Technology database 62 (NIST-62).

### Insects and plants used

The toxicity of *C. longa* to *T. ni* was initially evaluated in the laboratory (22 ± 3 °C, 70 ± 5% RH and a 16:8-h L:D) and later in a greenhouse (28 ± 3 °C, 16:8-h L:D).

Cabbage loopers used in bioassays were obtained from the Great Lakes Forestry Centre (GLFC), Canadian Forest Service (CFS), in Sault Ste. Marie, Canada. Approximately 25 eggs were introduced per plastic cup (50 mL) with an artificial diet McMorran^®^ [ingredients: agar, alphacel, ascorbic acid, aureomycin, casein, formaldehyde, linseed oil from flax plant (*Linum usitatissimum* L., Malpighiales: Linaceae), methyl paraben, potassium hydroxide, sugar, vitamins, water, wheat germ, and Wesson salt]. Cabbage plants (*B. oleraceae* var. Stonehead) used in the greenhouse bioassays were grown in plastic pots with a mixture of sandy loam soil and peat moss (4:1).

### Extraction and structural characterization of *ar*-turmerone

Rhizomes of *C. longa* were air-dried at 40 °C for three days and ground into a fine reddish-yellow powder (turmeric powder). The major chemical constituents of turmeric powder are curcuminoids, including bisdemethoxycurcumin (Fig. 2D), curcumin (3.14%) (Fig. 2E) and demethoxycurcumin (Fig. 2F)^58^. Other general constituents include proteins, resins and sugars^59^. Some volatile components could be lost by drying the rhizomes at 40 °C. However, our objective was to test the non-volatile components in the present study. Moreover, *ar*-turmerone has a high molecular mass and is not volatile at 40 °C.

An aliquot of the turmeric powder was reserved for bioassays and the part of the remainder extracted by steeping in hexane freshly distilled at 25 ± 3 °C with occasional stirring for a period of six hours. Five hundred grams of rhizome powder was extracted with 1 L hexane. The solution obtained was filtered and the solvent removed in a rotary evaporator under low pressure, yielding a light-yellow oil (=crude essential oil). Some volatile compounds in turmeric crude essential oil include atlantone (Fig. 2G), turmerone (Fig. 2H) and zingiberene (Fig. 2I).

**Figure 3:**
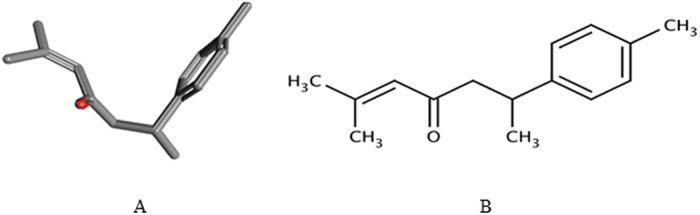
Chemical structures of *ar*-turmerone from *Curcuma longa* (Zingiberaceae) rhizomes, in 3D (**A**) and in 2D (**B**) conformers.

An aliquot of the crude essential oil was reserved for bioassays and the remainder was separated by column chromatography on silica gel (Vetec, 60–270 mesh), eluted with hexane:ethyl acetate (9:1). The fractions of interest, containing *ar*-turmerone (Fig. 3A,B), were analyzed by thin-layer chromatography (0.20 mm thickness, 60-mesh silica gel; Macherey-Nagel) visualized with iodine vapor (sublimation) and compared with a previously isolated and identified standard.

The other portion of the turmeric powder was separated by column chromatography on silica gel (Vetec, 60–270 mesh), eluted with hexane:ethyl acetate (1:1), to obtain curcuminoid pigments. These consisted of a mixture of bisdemethoxycurcumin (Fig. 2D), curcumin (Fig. 2E) and demethoxycurcumin (Fig. 2F)^60^.

### Contact toxicity (topical application) of turmeric powder and derivatives on cabbage loopers in the laboratory

Contact toxicity (measured as 24–48 h mortality) of turmeric powder and its derivatives with or without synergists was determined by topical application to early third instar *T. ni* following previous methodology with slight modifications^8^,^61^. Each larva received 1 μL of acetonic solution of turmeric powder or derivatives (dose = 10 μg per 3^rd^ instar cabbage looper) or a 1:1 binary mixture with one of the synergists (5 μg of turmeric powder or its derivatives +5 μg of PBO or other synergists), on the dorsum with a repeating dispenser attached to a 50 μL syringe. Acetone and Shultz Insect Spray^®^, alone, were used as negative and positive controls, respectively. After the compounds were applied, the larvae were transferred to Petri dishes (90 mm diameter × 15 mm height) in groups of 10 along with a small piece of artificial diet (1.12 g). There were three replicates of 10 larvae each per treatment. Treatment groups were placed in sealed plastic boxes lined with moistened paper towels and held for 48 h in a growth chamber (22 ± 3 °C, 16:8-h L:D). The mortality of cabbage loopers was determined after 24 and 48 h. Larvae were considered dead if they did not respond to prodding with forceps according to methodology described for velvetbean caterpillar, *Anticarsia gemmatalis* (Hübner, 1818) (Lepidoptera: Noctuidae) larvae treated with neem oil^14^. The LD_50_ (lethal dose causing 50% mortality) value was determined for treatments demonstrating >50% mortality at the initial screening concentration of 10 μg/larva. Mixtures were tested in a 1:1 ratio (dose = 5 μg of turmeric powder or its derivatives +5 μg of PBO or other synergists). LD_50_ values were calculated using the software EPA Probit Analysis Program, version 1.5^62^.

### Growth inhibitory effects of turmeric powder and derivatives on cabbage loopers in the laboratory

The effect of turmeric powder and its derivatives was assessed following modified methodology^63^. Acetonic solutions of the samples (20 mg/mL) were admixed with 3.5 g of dry artificial diet and allowed to dry in a fume hood for approximately 30 min. Following evaporation of the solvent, the artificial diet was mixed with an agar solution (0.5 g agar + 16 mL water were boiled and cooled before mixing to prevent the loss of compounds) to produce 20 g of treated artificial diet (1,000 ppm fwt). The negative control was an artificial diet prepared with acetone alone and the positive control contained Shultz Insect Spray^®^. Each 20 g piece of artificial diet was cut into 20 equal sized pieces and placed into individual compartments (3.81 cm length × 4.44 cm width × 2.54 cm depth) of plastic molded transparent assay trays (BIO-RT-32, C-D International, Pittman, NJ) and covered with perforated lids (Bio-CV4, C-D International, Pittman, NJ). One freshly hatched neonate larva (24 h old) was placed into each compartment (n = 20). The plastic trays with larvae were transferred into a clear plastic box (39.0 cm length × 27.0 cm width × 14.0 cm height) lined with a moistened paper towel and the box was placed in a growth chamber (24 ± 1 °C, 16:8-h L:D photoperiod, 70 ± 5% RH).

After seven days, all larvae were removed from the trays and individually weighed. The mean larval weight from each treatment was expressed as a percentage of the controls. The EC_50_ (effective concentration reducing larval growth by 50%) was determined using four concentrations of each sample (250, 500, 750 and 1,000 ppm fwt). Synergists were added at 1:1,000 part of the mixture (v/v).

Our contact toxicity experiments demonstrated high mortality in binary mixtures (turmeric powder or its derivatives + synergist in a 1:1 ratio) and, therefore, we could not use this ratio for growth inhibition experiments. Effect of an insecticide on the growth of larvae is normally assessed at 1,000 ppm. Synergists tested alone at this concentration did not cause any growth inhibition or mortality of the larvae and were therefore not included in statistical analysis.

### Protection of cabbage leaves treated with turmeric powder or its derivatives with or without synergists in the laboratory

Five cabbage leaves were treated with 160 μL of acetonic solution of turmeric powder or one of its derivatives with or without synergists (PBO, piperonal or sesamol). Solutions were applied and carefully dispersed over the entire area of the leaves using a pipette. Larvae (third-instar) were introduced onto each leaf and allowed to feed for four days. There were five replicates per treatment with six insects per replicate. Larvae collected from each leaf were counted and weighed on day four. Individual treatments were tested at 1% and mixtures in a 1:1 ratio of each constituent. Shultz Insect Spray^®^ (diluted to 1%) and acetone were used as positive and negative controls, respectively.

Shultz Insect Spray^®^ was used as a positive control because it is based on pyrethrins that are both toxic and inhibit growth of cabbage looper larvae. It was tested at 1,000 ppm in the artificial diet and 10 μg/larva in contact toxicity bioassays.

### Protection of intact cabbage plants treated with turmeric powder and derivatives in the greenhouse

We modified previous methodology^8^,^61^ to demonstrate the protection of intact cabbage plants in the greenhouse. Cabbage plants were grown individually in plastic pots over five to six weeks in the greenhouse until the six to eight leaf stages. Plants were then removed from their trays and arranged into three groups of five plants. All groups were sprayed with treatments until runoff with a hand-held sprayer. Treatments consisted of 1% acetonic solutions of turmeric powder or its derivatives with or without PBO (1:1 ratio). Acetone was used as a negative control and Shultz Insect Spray^®^ (1%) as a positive control.

The plants were air-dried, and five third instar cabbage loopers were introduced onto each plant (n = 75 larvae per treatment). The plants were randomly placed on a table under grow lights (400-Watt light bulbs per square meter) in the greenhouse (28 ± 3 °C, 16:8-h L:D photoperiod). On day four, larvae were removed from the plants, placed in separate polystyrene cups, and weighed in the laboratory. Experiments were repeated twice.

### Statistical Analysis

Mortality data were subjected to Probit analysis to determine LD_50_ values (lethal dose causing 50% mortality) and their corresponding 95% confidence intervals using the EPA Probit Analysis Program version 1.5. LD_50_’s are considered significantly different from one another if their 95% confidence intervals (C.I.) do not overlap. EC_50_ (effective concentration reducing larval growth by 50%) values were calculated by using linear regression analyses in Microsoft Excel. Growth inhibition data were analyzed using the Statistics 7 program for analysis of variance (ANOVA). When significant F values were found, Tukey’s HSD multiple-comparison tests were used to test for significant differences between individual treatments. Experiments were repeated at least twice.

## Additional Information

**How to cite this article**: de Souza Tavares, W. *et al.* Turmeric powder and its derivatives from *Curcuma longa* rhizomes: Insecticidal effects on cabbage looper and the role of synergists. *Sci. Rep.* **6**, 34093; doi: 10.1038/srep34093 (2016).

**Publisher’s note:** Springer Nature remains neutral with regard to jurisdictional claims in published maps and institutional affiliations.

## Figures and Tables

**Table 1:** 

Treatments	24 h mortality (%) ± SE^1^
Turmeric crude essential oil	10.0 ± 0.00
Turmeric powder	10.0 ± 0.00
*ar*-Turmerone	20.0 ± 5.70
Curcuminoid pigments	16.7 ± 3.30
Turmeric crude essential oil + Piperonal	10.0 ± 0.00
Turmeric powder + Piperonal	16.6 ± 3.30
*ar*-Turmerone + Piperonal	23.3 ± 0.40
Curcuminoid pigments + Piperonal	6.60 ± 0.33
Turmeric crude essential oil + Sesamol	10.0 ± 0.00
Turmeric powder + Sesamol	6.60 ± 0.33
*ar*-Turmerone + Sesamol	16.6 ± 3.30
Curcuminoid pigments + Sesamol	8.00 ± 0.33
Turmeric crude essential oil + PBO	90.0 ± 7.20
Turmeric powder + PBO	94.0 ± 5.40
*ar*-Turmerone + PBO	96.7 ± 3.60
Curcuminoid pigments + PBO	33.3 ± 15.8
Positive control (Shultz Insect Spray^®^)	71.4 ± 13.3

Toxicity of turmeric powder and its derivatives from *Curcuma longa* (Zingiberaceae) rhizomes with or without synergists against third instar cabbage looper, *Trichoplusia ni* (Lepidoptera: Noctuidae) at an initial screening concentration in the laboratory. n = 3 × 10 insects per treatment. Turmeric crude essential oil, turmeric powder, *ar*-turmerone, and curcuminoid pigments were tested at 10 μg/larva. Mixtures of turmeric powder or its derivatives + synergists were tested in a 1:1 ratio of each constituent (dose = 5 μg + 5 μg/larva). PBO = Piperonyl butoxide. Shultz Insect Spray^®^ was tested at 10 μg/larva.

^1^Standard error. Synergists alone did not cause any mortality at the dose tested (5 or 10 μg/larva) and are therefore not included in the analysis. Mortality of cabbage looper larvae was <10% for individual treatments of turmeric crude essential oil, turmeric powder, *ar*-turmerone, and curcuminoid pigments at a dose of 5 μg/larva (data not reported). We decided to use 1:1 ratio in binary mixtures as this is a common practice in our laboratory. PBO is usually added in a ratio of 10:1 in many commercial insecticides; we have made a simple combination consisting of equal parts of each constituent.

**Table 2:** 

	*ar*-Turmerone	Oil	Pigments	Powder
LD_50_ (μg/larva)^1^ (95% CI)^2^	0.26 (0.14–0.38)	0.05 (0.03–0.08)	0.61 (0.447–1.00)	0.03 (0.00–0.11)
χ^2^	4.90	4.93	5.9	1.19
Slope ± SE^3^	1.45 ± 0.29	1.45 ± 0.29	1.82 ± 0.39	0.52 ± 0.13

Dose response effect of binary mixtures of turmeric powder (‘Powder’) and derivatives [*ar*-turmerone, turmeric crude essential oil (‘Oil’) and curcuminoid pigments (‘Pigments’)] from *Curcuma longa* (Zingiberaceae) rhizomes with the synergist piperonyl butoxide (PBO) at a 1:1 ratio against third instar cabbage looper, *Trichoplusia ni* (Lepidoptera: Noctuidae) via topical application. n = 3 × 10 insects per treatment.

^1^Concentration causing 50% mortality. LD_50_ values were based on 4–5 concentrations (0.01–10 μg/larva).

^2^Confidence interval.

^3^Standard error. Mixtures (turmeric powder and its derivatives + PBO) were tested in a 1:1 ratio. Chi-squared test is measuring the null hypothesis that the slope is zero.

**Table 3:** 

Treatments	Mean (mg) ± SE^1^	Losses (%)	Reduction (%)
Turmeric powder	196.5 ± 20.6 b	66.0	34.0
Turmeric crude essential oil	178.6 ± 24.5 bcd	60.0	40.0
*ar*-Turmerone	119.1 ± 23.8 ef	40.0	60.0
Turmeric powder + Piperonal	202.9 ± 20.4 b	67.9	32.1
Turmeric crude essential oil + Piperonal	207.8 ± 22.3 b	69.6	30.4
*ar*-Turmerone + Piperonal	135.6 ± 34.3 de	45.5	54.5
Turmeric powder + Sesamol	172.3 ± 26.7 bcd	57.8	42.2
Turmeric crude essential oil + Sesamol	214.8 ± 26.7 b	71.9	28.1
*ar*-Turmerone + Sesamol	116.7 ± 25.6 ef	39.0	61.0
Turmeric powder + PBO	190.9 ± 29.2 bc	64.1	35.9
Turmeric crude essential oil + PBO	144.0 ± 25.2 cde	48.5	51.5
*ar*-Turmerone + PBO	81.80 ± 19.4 f	27.5	72.5
Negative control (Acetone)	297.8 ± 29.5 a	—	—
Positive control (Shultz Insect Spray^®^)	200.0 ± 28.7 b	64.8	35.2

Growth inhibition of cabbage looper, *Trichoplusia ni* (Lepidoptera: Noctuidae) larvae on artificial diets containing turmeric powder and its derivatives from *Curcuma longa* (Zingiberaceae) rhizomes and binary mixtures with three synergists evaluated with the parameters: mean weight of insects (‘Mean’) (mg), mean weight losses compared to the control (‘Losses’) (%) and mean weight reduction (‘Reduction’) (%). Turmeric powder and its derivatives were treated at 1,000 ppm. Mixtures (turmeric powder and its derivatives + synergists) were tested at 1:1,000 part of the diet. N = 19–20 insects per treatment. PBO = Piperonyl butoxide. Shultz Insect Spray^®^ contains 0.02% pyrethrins and 0.20% PBO.

^1^Standard error.

**Table 4:** 

Treatments	EC_50_ (ppm)^1^	
	Without PBO (R^2^)^2^	With PBO (R^2^)^3^
*ar*-Turmerone	608.7 (0.87)	304.7 (0.86)
Turmeric crude essential oil	844.4 (0.88)	598.9 (0.73)
Turmeric powder	765.3 (0.88)	471.2 (0.88)

Dose response effects of binary mixtures of turmeric powder and its derivatives from *Curcuma longa* (Zingiberaceae) rhizomes with piperonyl butoxide (PBO) on growth of cabbage looper, *Trichoplusia ni* (Lepidoptera: Noctuidae) larvae through feeding on an artificial diet. n = 20 larvae per treatment.

^1^Effective concentration causing 50% growth inhibition. EC_50_ values were based on 4–5 concentrations (125–1,000 ppm).

^2^Coefficient of determination.

^3^1:1,000 part (PBO:treatment). Positive control (Shultz Insect Spray^®^, containing 0.02% pyrethrins and 0.20% PBO) demonstrated 35% reduction in weight at 1,000 ppm.

**Table 5:** 

Treatments	Mean (mg) ± SE^1^	Relative (%)	Reduction (%)	Recovered
Negative control (acetone)	46.1 ± 20.4 a	—	—	25
Turmeric crude essential oil	35.9 ± 19.4 ab	78	22	20
Positive control (Shultz Insect Spray^®^)	35.2 ± 18.5 ab	76	24	20
Turmeric powder	33.8 ± 18.6 ab	73	27	21
Turmeric powder + PBO	33.6 ± 18.7 ab	73	27	17
Turmeric crude essential oil + PBO	31.6 ± 17.9 ab	69	31	16
*ar*-Turmerone	24.3 ± 11.4 bc	53	47	13
*ar*-Turmerone + PBO	14.5 ± 7.60 c	31	69	09

Effects of turmeric powder or its derivatives from *Curcuma longa* (Zingiberaceae) rhizomes with or without piperonyl butoxide (PBO) on cabbage looper, *Trichoplusia ni* (Lepidoptera: Noctuidae) larvae in the laboratory applied on cabbage, *Brassica oleracea* var. Stonehead (Brassicaceae) leaves using a pipette and evaluated with the parameters: mean weight of insects (‘Mean’) (mg), mean weight of the insect relative to the control (‘Relative’) (%), mean weight reduction (‘Reduction’) (%), and number of larvae recovered (‘Recovered’). Means followed by the same letter do not differ significantly (Tukey’s test, p < 0.05). Five leaves were used per treatment. n = 6 larvae per leaf (N = 30 larvae per treatment). Individual treatments and the positive control were tested at 1%. Mixtures were tested in a 1:1 ratio. Shultz Insect Spray^®^ contains 0.02% pyrethrins and 0.20% PBO.

^1^Standard error.

**Table 6:** 

Treatments	Mean (mg) ± SE^1^	Relative (%)	Reduction (%)	Recovered
Negative control (acetone)	49.5 ± 19.4 a	—	—	50
Turmeric crude essential oil	36.9 ± 19.1 ab	74.5	25.5	46
Positive control (Shultz Insect Spray^®^)	35.2 ± 19.4 bc	71.1	28.9	43
Turmeric powder	33.9 ± 16.2 bc	68.4	31.6	45
Turmeric powder + PBO	29.9 ± 19.3 bc	60.4	39.6	33
Turmeric crude essential oil + PBO	22.6 ± 11.5 cd	45.6	50.4	32
*ar*-Turmerone	15.1 ± 05.4 d	30.5	69.5	30
*ar*-Turmerone + PBO	14.6 ± 03.5 d	29.5	70.5	19

Effects of turmeric powder or its derivatives from *Curcuma longa* (Zingiberaceae) rhizomes with or without piperonyl butoxide (PBO) on cabbage looper, *Trichoplusia ni* (Lepidoptera: Noctuidae) larvae in the laboratory applied on cabbage, *Brassica oleracea* var. Stonehead (Brassicaceae) leaves using a pipette and evaluated with the parameters: mean weight of insects (‘Mean’) (mg), mean weight of the insect relative to the control (‘Relative’) (%), mean weight reduction (‘Reduction’) (%), and number of larvae recovered (‘Recovered’). Means followed by the same letter do not differ significantly (Tukey’s test, p < 0.05). Three replicates with five plants each were used (n = 5 larvae per plant; n = 75 larvae per treatment). Individual treatments and the positive control were tested at 1% acetonic solutions. Mixtures were tested in a 1:1 ratio. Shultz Insect Spray^®^ contains 0.02% pyrethrins and 0.20% PBO.

^1^Standard error. Shultz Insect Spray^®^ was diluted to match the concentration of our treatments and therefore did not meet expected efficacy against cabbage looper.
